# The Collective Investigation of Disease

**Published:** 1884-01

**Authors:** 


### Editorial.

### ARTICLE X

THE COLLECTIVE INVESTIGATION OF DISEASE.

There are many points upon which doctors differ, as those who are not doctors have long since sarcastically remarked. But there are also many points upon which doctors are quite agreed, an observation to which those who are not doctors give very little heed. The relative proportion existing between the two, facts disputed and facts accepted, it is not easy to determine. All, however, are quite in accord respecting the pressing need of enlarging the proportion borne by the latter to the former, respecting the lamentable doubt which enshrouds too many facts often held to be indisputable as to proof, and respecting the always formidable disproportion between that which is actually known partly or fully, and the great unknown.

At no time in the history of the world has there been an age when, as compared with the present, the investigations of science have been more fearlessly pushed and the results more calmly weighed and more judiciously compared. At the same time it must be conceded that the net profit has been inferior in value and extent to that which might have been gained by the systematic efforts of the same investigators working with a harmony of purpose and method. The individual and isolated success of the man who discovers a "nugget" is far surpassed by the gross rewards of the company that exhausts a "vein."

These remarks are suggested by the general interest lately awakened in the subject of the collective investigation of disease, as this has been undertaken in England chiefly, but also it appears in the United States, in Germany and some parts of India. This investigation of certain special subjects connected with disease is conducted by the distribution of cards, whose blank spaces are to be filled with the data which it is desired to accumulate either touching certain specially selected diseases, or certain selected features of disease, or the action of certain remedies. The filling up of the blanks on these cards by separate observers and a subsequent collation of the accumulated facts, offer a most attractive prospect for the future progress of medical science. The late addresses upon this interesting subject, of the distinguished Professor of Pathology and Surgery in the Royal College of Surgeons, Mr. Jonathan Hutchinson, and of Dr. William Roberts, of Owen's College, before the Lancashire and Cheshire branch of the British Medical Association, together with the debate which they naturally suggested, present with clearness the advantages to be thus gained and some of the obstacles to be overcome.

Fortunately or unfortunately, the obstacles have usually to be first surmounted, and, therefore demand an early consideration. Not the least among these springs from the disparity in the attainments of the several colaborators. Medical instructors know only too well how difficult it is to teach men habits of accurate observation. But observation is not enough. The three-fold task of observation, record, and interpretation, is required of all scientific investigators. Some men fail in one, and some in another part of this important process. Thus in face of a fracture one man distinguishes with his eyes an ecchymosis and a helpless limb, but by his sense of touch fails to recognize any crepitus. He is sufficiently ignorant to decide against the possibility of fracture, not by reason of what he has actually seen but by reason of what he has failed to perceive by the touch. Another, equally ill-educated, observes the ecchymosis, makes sufficient record of it mentally, and failing to observe the other symptoms, diagnosticates a rupture of a blood vessel. The degree of education requisite to properly observe may be said to have no definite limit. What we have daily spread before our eyes and fail to interpret, makes up a world into which we never enter, so large that it has fired the zeal of all honest explorers since science was first cultivated among men.

Aside from the inevitable ignorance of many men, or to put it on a truer basis. the different grade of their attainments, there are obstacles in all such work to be found, in the carelessness and even the desire for notoriety which leads some weak men to report what they have never seen. Then rises another difficulty which Mr. Hutchinson wisely describes in advance, one growing out of an inevitable tendency to unduly multiply the points upon which information is wanted. In the diversity of these and their confusing tendencies, an attempt is made to do too much which results in doing nothing at all. It is one of the most difficult of lesions to learn, to cover patiently and faithfully a small corner of a wide field. Few have the indefatigable perseverance of a Darwin, to count the weeds that grow in a square inch of soil and the several varieties of insects that will visit it on a summer day.

Now, in the face of these and other adverse circumstances, it is pleasant to note that the results actually obtained have surprised all who have examined them, not merely those who were interested in the experiment, but even those who went into the examination of the returns with a despondent and gloomy opinion as to the fruit likely to be obtained. It has happened that the poorer class of men neither felt an interest in the work, nor exhibited any, while the returns were found to have been forwarded by the very best men in the several branches of the British Medical Association. Dr. Mahomed, in discussing the two addresses, declared that men had come to him and expressed their astonishment at the extent and valuable character of the material which had been accumulated; that it was evidently the work of men who had studied their cases, and recognized and recorded the facts upon the points required with perfect accuracy and with trustworthy results. The reporters on pneumonia, for instance, said that they believed there was not such a mass of information in existence in any other language as had been reported, and it would give rise to the most important monograph on the subject hitherto written, giving actual facts and statistical details about this disease.

Dr. Ransome, in his discussion of this same subject, declared that the results obtained were " quite unequaled, and would form in the future a mine of information on the subject, not only of epidemics, but other diseases."

Dr. Roberts, referring to the admirable report on the communicability of phthisis, drawn up by Dr. Burney Yeo, declared that no candid person, after comparing the report with the detailed evidence upon which it was based, could fail to conclude that a healthy person should not be permitted to occupy the same bed with a sufferer from pulmonary consumption; and that no person with an hereditary predisposition to tuberculous disease should be allowed to have continued and intimate personal contact with a phthisical patient. The speaker went on to say that, in his opinion, a grave responsibility would be incurred by a medical man possessed of this evidence, no matter what his theoretical opinions might be, who would permit such cohabitation and close personal contact.

Some of the issues and subordinate questions brought to the front in the addresses and subsequent debate are suggestive—some rather amusing—yet others little likely to be of any practical value in the end.

Thus, Dr. Roberts suggests that one of the most promising fields of labor for combined observation, is the self-study of disease by medical men. He admits that it would be rather a tragical task for the physician affected with a fatal malady to make a study of his serious symptoms, and suggests "the dyspepsia of healthy persons" (*sic*) as a fertile field, in the belief which he expresses, that at least one of ten of the ten thousand members of the British Medical Association is an habitual dyspeptic of one type or another. These remarks, it must be confessed, sound rather oddly to us on this side of the Atlantic. Our British friends have so often charged us with being a nation of dyspeptics, that we should expect to find our medical brothers exhibiting their due proportion of such a disease, but we are quite confident that there is nothing like such a ratio of dyspepsia among American physicians.

But, we do not believe that the best information respecting any disease will ever be gained by physicians who are its victims. They who have had much to do with the treatment of medical men, have been taught that in proportion as a doctor becomes seriously ill, he departs from the professional plane and approaches that of the most unlearned of patients. Introspection, self study, self observation, may suffice for the intricate absurdities of certain schools of philosophy, but, like solitary vices, they are not promising methods for the accumulation of scientific facts.

Again, Mr. Hutchinson's view that the rarer and curious maladies should be especially selected for collective investigation, is certainly worthy of consideration. To know the rule, it is certainly necessary to understand all its exceptions. There are few observers of large experience who have not again and again become convinced of the fact that what is generally deemed to be new and rare is often indeed old and common but unrecognized; and that the maladies of men, in all ages and in all climates, are as kindred to each other as are the races of mankind themselves. But still we must admit that the list of rare diseases presented by Mr. Hutchinson is one containing the names of maladies so exceedingly rare that they may never be encountered in a generation of active practice. Such, for example, are rhinoscleroma, Hodgkin's disease, Paget's osteitis deformans, and Charcot's joint disease. It is difficult to imagine that even twenty-five per cent. of all the medical men to whom the results became accessible would be greatly better for the observations of four thousand of their studious brethren upon these points. We can imagine, however, that a much larger proportion would very much like to have the results obtained by even one thousand skilled observers upon, for example, the significance of symmetrical contracture of the digits in diseases of the cerebro-spinal tract, or upon the characters which distinguish the several varieties of lumbar pain in nephritis, peri-nephritic abscess, muscular rheumatism, certain of the exanthemata, menstrual disorders, and over exertion. They certainly would be interested in knowing the effects of the several medicaments recognized as valuable in spasmodic asthma, after the administration of each in say two hundred similar cases. In some such homely investigations of disease it is possible that there might be awakened a livelier interest, by the anticipation of results which could be made more immediately and practically useful.

Still, it is far more easy to criticise than to commend, and in the present case certainly commendation alone is required. We take this opportunity of directing especial attention to this important subject, with a view to more widely interesting the medical men of our own country in the same fruitful field. They who are in the forefront of the unresting scientific activities of our day see that the immediate future of medical knowledge involves very remarkable and radical changes. It is about to rest on many more established facts, with much less speculation in all the elements of its structure, and with the plucking out of enormous quantities of effete material, with regard to which it may be questioned whether it has ever been of even temporary advantage. The collective investigation of disease, simultaneously conducted in different countries, by men of intelligence, with the proper training, experience, and opportunities, is certainly one of the means by which this end is to not merely to be gained, but is actually in process of attainment.

